# Impact of Lifestyle Medicine Interventions on the Management of Systemic Hypertension in Primary Care: A Canadian Randomized Controlled Trial

**DOI:** 10.1177/15598276241242013

**Published:** 2024-04-04

**Authors:** Elisa Marin-Couture, Julie-Alexandra Moulin, Anne-Sophie Thibault, Paul Poirier, Jean-Pierre Després, Anette Gallant, Vincent Lamarre, Natalie Alméras, Isabelle Lemieux, Christian Chabot, Maria-Cecilia Gallani, Marie-Eve Piché, Benoit J. Arsenault, Angelo Tremblay, Jean-Sébastien Paquette, Caroline Rhéaume

**Affiliations:** 1 Department of Kinesiology, Faculty of Medicine, 4440 Université Laval, Québec, QC, Canada (EMC, JPD, NA, AT); 2 Centre de Recherche Nutrition, Santé et Société (NUTRISS), INAF, 4440 Université Laval, Québec, QC, Canada (EMC, AT); 3 VITAM – Centre de Recherche en Santé Durable, Québec, QC, Canada (EMC, JAM, JPD, CC, JSP, CR); 4 Family Medicine Teaching Unit (Groupe de Médecin de Famille Universitaire: GMF-U Quatre Bourgeois), Québec, QC, Canada (AST, CR); 5 Department of Family Medicine and Emergency Medicine, Faculty of Medicine, 4440 Université Laval, Québec, QC, Canada (AST, JSP, CR); 6 Centre de Recherche de l’Institut Universitaire de Cardiologie et de Pneumologie de Québec, 4440 Université Laval, Québec, QC, Canada (PP, JPD, AG, VL, NA, IL, MCG, MEP, BJA, AT, CR); 7 Faculty of Pharmacy, 4440 Université Laval, Québec, QC, Canada (PP); 8 Faculty of Nursing, 4440 Université Laval, QC, Canada (MCG); 9 Department of Medicine, Faculty of Medicine, 4440 Université Laval, QC, Canada (BJA); 10 Primary Care Research and Innovation Laboratory (Laboratoire ARIMED), Groupe de Médecine de Famille Universitaire du Nord de Lanaudière, Joliette, QC, Canada (JSP)

**Keywords:** hypertension, lifestyle medicine, primary care, medication

## Abstract

The study aimed to evaluate the feasibility of implementing lifestyle interventions in primary care settings with hypertensive patients and their effect on blood pressure, body composition, cardiometabolic markers, and antihypertensive drug use. Sixty participants diagnosed with stage 1 hypertension were randomly assigned to 4 groups: (1) Standard medical care (control), (2) Physical activity protocol, (3) Dietary Approach to Stop Hypertension (DASH) diet, and (4) Combination of physical activity protocol and DASH diet. Participants received counseling from family physicians, nurses, kinesiologists, and registered dietitians. Various assessments were conducted before (T0) and after (T6) the interventions, including 24-h ambulatory blood pressure monitoring, blood and urine tests, anthropometric measurements, computed tomography to measure adipose tissue, submaximal exercise test to estimate maximal oxygen consumption and health questionnaires. Fifty-one (51) participants (51/57, 89%) completed the program. All interventions reduced blood pressure indices between T_0_ and T_6_, except the combined interventions group. Body composition and cardiometabolic parameters were improved in all groups, except for the control group. In total, 28% of participants (7/23) reduced or stopped their antihypertensive medications at T_6_. The results suggest that structured lifestyle interventions are feasible in primary care and improve blood pressure and cardiometabolic parameters in patients with stage 1 hypertension.

“The study aimed to evaluate the feasibility of implementing lifestyle interventions in primary care settings with hypertensive patients and their effect on blood pressure, body composition, cardiometabolic markers, and antihypertensive drug use.”

## Introduction

Systemic hypertension (HTN) has been recognized as the main modifiable risk factor leading to cardiovascular diseases (CVD) and all-cause mortality worldwide.^1^ According to Statistics Canada, approximately 25% of Canadian adults are considered hypertensive.^2^ High blood pressure is known to be influenced by physical inactivity,^3,4^ low cardiorespiratory fitness (CRF),^5^ a diet low in fruits and vegetables,^6^ high sodium intake,^7^ being overweight or obese,^8^ especially high levels of visceral adipose tissue (VAT),^8,9^ and the presence of type 2 diabetes and chronic kidney disease.^2^ Cheng et al^10^ recently conducted a meta-analysis that demonstrated that individuals with higher CRF had a 37% lowered risk of HTN compared to individuals with low CRF. They also showed a stronger protective effect of CRF on HTN in lean individuals [body mass index (BMI) < 25 kg/m^2^] than in their overweight/obese counterparts. These findings are also in agreement with results of our laboratory demonstrating the relationship between the independent contributions of VAT accumulation and poor CRF on elevated systolic (SBP) and diastolic blood pressure (DBP).^11,12^

The International Society of Hypertension,^13^ the American Medical Association,^14^ and Hypertension Canada^15^ have revised their guidelines to provide improved guidance to healthcare professionals in the management and treatment of HTN.^16^ They emphasize that lifestyle interventions and/or prescription of antihypertensive medications are both primary treatments for individuals with HTN. They promote the initiation of healthy lifestyle behavior changes as the primary approach, prioritizing them over antihypertensive treatment. They encourage patients to adopt a healthy lifestyle, even when hypertension medication is prescribed. In this regard, Unger et al^13^ reported that adopting a healthy lifestyle serves as a preventive measure against HTN, consequently lowering the risk of CVD. Notably, the European Society of Cardiology/European Society of Hypertension (ESC/ESH) places significant emphasis on lifestyle interventions as the sole recommended treatment for individuals with stage 1 HTN (SBP/DBP = 140-159/90-109 mm Hg) during the initial 3-6 months post-diagnosis. Medications are only advised if HTN is inadequately controlled beyond this specified period.^17^ It is noteworthy that American College of Cardiology and the American Heart Association AHA’s have different cutoffs for defining stage 1 HTN compared to the ESC/ESH guidelines (SBP/DBP = 130-139/80-89 mm Hg),^18^ potentially resulting in more patient being prescribed antihypertensive drugs.

Moreover, a substantial proportion of healthcare costs, approximately 80%, is attributable to chronic diseases.^19^ Equally, lifestyle factors are known to be associated with the development of chronic diseases.^20^ Despite this ample evidence demonstrating the positive impact of lifestyle interventions in HTN management, their implementation in primary care practice remains limited, and only a few studies have been conducted in such settings.^21^ Lifestyle medicine (LM) is an emerging field within medicine and primary care and family medicine serve as natural domains for LM expertise.^22^ Indeed, in this study, we aimed (1) to evaluate the feasibility of implementing a 6-month interdisciplinary lifestyle program among hypertensive patients, focusing on changes in SBP and DBP in primary care settings, (2) to investigate the relationship between changes in SBP and DBP and variations in body composition and cardiometabolic markers, and (3) to document the changes in SBP, DBP, and cardiometabolic parameters in hypertensive patients receiving antihypertensive medications compared to those who were not medicated for their HTN.

## Methods

### Study Population

Patients (n = 124) were referred by their family physicians or allied healthcare providers between February 2013 and December 2016 to the Academic Family Medicine Group (GMF-U QB), affiliated with Université Laval. They all underwent 24-h ambulatory blood pressure monitoring (ABPM) to confirm their HTN status. Participants of the present study had to meet the following criteria: (1) to be aged >18 years old; (2) to be diagnosed with stage 1 HTN^23,24^ confirmed with the use of a 24-h ABPM, treated or not with antihypertensive medication(s); (3) Any treated medical conditions needed to be stable last for 3 months, that is, no change in health status or medication use; and (4) to reach <10,000 daily steps measured by a wearable tracker (Timex T5E011, Middlebury, CT USA). Smokers and patients with secondary HTN, and other comorbidities (i.e., type 1 and type 2 diabetes, dyslipidemia according to the Canadian Cardiovascular Society,^25^ glomerular filtration rate <30 mL/min/1.73 m^2^), or CVD (arrhythmia, heart valve disease, myocardial infarction, angina pectoris, etc.) were excluded. Following the initial 24-h ABPM evaluation, 64 patients were excluded for displaying normal blood pressure (BP) indices values. Therefore, 60 patients with stage 1 HTN were included and consented to participate in the study (non-medicated patients: n = 35; medicated patients: n = 25).

### Ethical Consideration

The authors are accountable for all aspects of the work. The trial was conducted following the principles of the Declaration of Helsinki.^26^ Ethics approval was obtained from the Institutional Ethics Board of the Centre intégré universitaire de santé et de services sociaux (CIUSSS) de la Capitale-Nationale (NO.: 2012-2013-10) and l'institut de cardiologie et de pneumologie de (NO.: 20805). Signed informed consent was given by all participants before inclusion. The complete study protocol is a registered clinical trial (clinicaltrials.gov, ID: NCT02492035).

### Study Design

**Figure 1.**
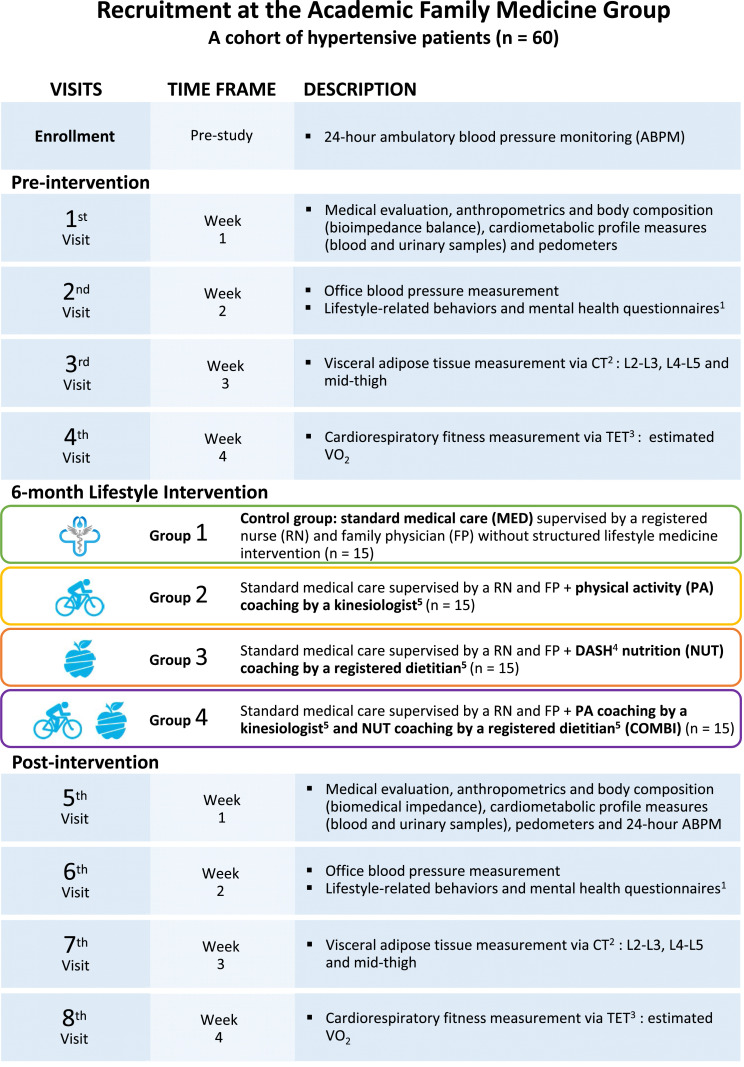
Study design and intervention details. Note: ^1^Questionnaires were Educoeur-en-route-77Q; physical activity assessment (3-day physical record); dietary assessment (3-day dietary record, Nutrition habit-27Q; Three-factor eating questionnaire-51Q (TFEQ), Beck Anxiety Inventory-21Q (BDI), State-trait Anxiety Inventory-20Q (STAI), SF-36-Q11 (quality of life); Food Frequency Questionnaire. Abbreviations: ^2^CT, computed tomography; ^3^TET, treadmill exercise test; ^4^DASH, Dietary Approaches to Stop Hypertension. ^5^Lifestyle intervention: all participants were meeting with a kinesiologist and/or a registered dietitian for 7 visits over 6 months, that is, 2 visits in the first month following by monthly visits for the 5 other months.

Figure 1 presents the study design and details of the interventions. This 6-month randomized pilot study was conducted in a primary care setting in Québec City (GMF-U QB). Eligible patients (n = 60) were randomized into one of the following intervention groups: (1) standard medical care (MED) which was considered the control group followed-up by family physicians and registered nurses and receiving dietary and PA recommendations aiming to reduce BP (n = 15), (2) physical activity protocol supervised by kinesiologist (PA) (n = 15), (3) Dietary Approach to Stop Hypertension (DASH) diet supervised by registered dietitian (NUT) (n = 15), and (4) a combination of both PA and NUT interventions supervised by both healthcare providers (COMBI) (n = 15). They underwent a series of assessments including medical evaluation, anthropometric measurements, computerized tomography scan measuring VAT distribution, blood and urine collection, submaximal exercise test, and health questionnaires, during the 4 weeks leading up to the intervention timeframe and at the 6-month mark. All assessments were consistently carried out in the same order to ensure reliability. The research coordinator played a pivotal role throughout the study. This role encompasses several key responsibilities, including reviewing consent forms with participants, facilitating appointment scheduling between healthcare providers and participants, and organizing interdisciplinary meetings. Google Drive was used for managing participant’s appointments.

### Lifestyle Medicine Intervention Structure

The intervention programs followed the Hypertension Canada guidelines—Canadian Hypertension Education Program^24^ and integrated some key elements of the Expanded Chronic Care model.^27^ The entire team of healthcare providers received training from the Société uébécoise d’hypertension artérielle (SQHA).^28^ Participants met with the healthcare providers related to their allocated lifestyle intervention twice during the first month and monthly for the subsequent 5 months. Participants randomized in the PA and the COMBI groups met with a kinesiologist who prescribed individualized fitness plans. The ultimate goal of the kinesiologist was to promote the SQHA’s recommendations, which included engaging in aerobic activities for at least 4 days a week with each session lasting 30 to 60 min, participating in a minimum of 2 strength training sessions per week,^29^ and improving daily steps to achieve 10,000 steps a day. Participants in the NUT and COMBI groups met with a registered dietitian to adapt their diet based on the DASH diet.^30,31^ Participants in the control group met with their family doctor at the beginning of the study and as needed during the study. All participants also met monthly with a registered nurse who measured vital signs, that is, office BP using an automated sphygmomanometer (Welch Allyn, Spot Vital Signs ® Device, Canada)^24^ and heart rate (HR), as well as body weight and waist circumference (WC). The registered nurse also provided teaching to the patient about lifestyle medicine and management of HTN with the SQHA’s recommendations. A patient partner was also part of our team. Their role typically involved actively participating in the healthcare decision-making process alongside healthcare providers and researchers, sharing experiences throughout the study, and providing feedback to advocate patient-centered care. Additionally, the entire healthcare team was meeting weekly to deliberate on participant progress, study procedures, collected data, interdisciplinary care coordination, and any necessary adjustments, ensuring the study’s smooth progression and participant adherence to their assigned interventions. At the end of the study, each participant received counseling from their respective healthcare providers, who proposed additional recommendations to further improve their lifestyle habits. Moreover, they were provided with a detailed health report indicating their own BP and cardiovascular risk factor variations.

### Outcome Measurements

#### Blood Pressure

A 24-h ABPM (ABPM, 90207, Spacelabs Healthcare, WA, United States)^32^ was performed during enrollment and post-intervention. A registered nurse was responsible for installing the ABPM and advised patients on the instructions to follow to ensure standardization and accuracy of measurements.^32^ The cuff was placed on the non-dominant arm. The device was set to record intermittent measurements of SBP and DBP at intervals of 20 min during the day and 60 min at night. The reports indicated the mean SBP/DBP for 24 h, in addition to daytime and nighttime averages. The dipping percentage of BP between daytime and nighttime was also calculated. The reports were analyzed either by the principal investigator of the study, a family physician expert in ABPM readings, or by a cardiologist at the Institut universitaire de cardiologie et de pneumologie de Québec—Université Laval (IUCPQ-ULaval). In the present study, the mean SBP/DBP for 24 h was used. In addition, office BP were recorded monthly.

#### Cardiometabolic Risk Markers

Collection of blood samples following a 12-h fast was performed by a registered nurse according to standardized procedures. In this study, various biological markers associated with cardiometabolic health were examined: fasting plasma lipoprotein-lipid profile (total cholesterol, low-density lipoprotein (LDL) cholesterol, high-density lipoprotein (HDL) cholesterol, and triglyceride levels), glycated hemoglobin (HbA1c).

#### Anthropometric Measurements and Computed Tomography Scan

Body weight was measured using a bioelectrical impedance analysis scale (InBody 520, InBody USA, Cerritos, CA, USA). Height was measured according to standard procedures without shoes, while heels, buttocks, and upper back were in contact with a wall-mounted stadiometer. BMI was calculated by dividing body weight (kg) by squared height (m^2^). WC was measured at the midpoint between the superior iliac crest and the last rib according to standardized procedures.^33^ Cross-sectional areas of adipose tissue of the abdomen and the thigh were obtained by computed tomography scan.^34^ Calculation of the partial volume of visceral and subcutaneous adipose tissue at L_2_-L_3_, L_4_-L_5_, and mid-thigh levels were subsequently estimated by an experienced research professional according to standardized procedures. A specialized software was used (SliceOmatic, Tomovision, Montréal, Québec, Canada) to conduct the imaging analysis. This study only considered the L_4_-L_5_ VAT volume.

#### Physical Activity Level

Physical activity levels were assessed using a 3-day activity record^35^ and a pedometer (Timex T5E011, Middlebury, CT USA) to measure daily step count. Participants were required to wear the pedometer for 7 consecutive days during the pre- and post-intervention periods. Daily and weekly averages were calculated to determine daily step count pre- and post-intervention.

#### Estimated Maximal Oxygen Consumption and CRF

According to standardized procedures, all participants performed a progressive submaximal exercise test on a Q65 treadmill linked to a Q4000 monitor (Quinton Instruments Co., Seattle, WA, USA) and an automatic sphygmomanometer (Tango + stress BP, SunTech Medical). Blood pressure and heart rate were monitored before and during the test. Participants were supervised by a kinesiologist and a physician throughout the test. Briefly, following a 3-min warm-up at a speed of 2.5 mph and 0% slope, the kinesiologist increased the speed and the slope, including a standardized step of 3.5 mph and 2% slope, to achieve between 70% and 80% of the predicted maximal HR calculated with the Astrand formula, which normally represents ∼150 bpm. Maximal oxygen consumption (V̇O_2max_) was estimated by extrapolating oxygen consumption to age-estimated maximal HR (220 - age)^36^ using the American College of Sports Medicine (ACSM)’s metabolic equations^37^ and the least square method. The CRF of participants was defined as the estimated V̇O_2max_.

#### Urinary Sodium

Collection of urine samples in fasting state was conducted in 27 patients, permitting the evaluation of the diet compliance.^38^ Using urinary sodium spots indicated if dietary changes were made for salt consumption reduction.

#### Medication

The family physician and registered nurse documented the medical history and medications. The family physician was also responsible for classifying and documenting changes in HTN medications. Medicated participants were classified at the end of intervention as followed: (1) no change, denoting case where HTN medication remained unchanged in both dose and prescription; (2) decrease, indicating patients whose HTN medication dosage was reduced or was no longer required as BP had reached normotensive target; and (3) increase, when HTN medication dosage was increased or when a new HTN medications was prescribed by the end of the intervention program. The principal investigator, a family physician, analyzed deprescription, which involved discussing medication prescriptions with the physician referring the patient. The use of over-the-counter drugs was also documented. In collaboration with the pharmacist, we developed a tool to inform the participant about which over-the counter drugs might increase blood pressure.

### Statistical Analyses

#### Sample Size

A sample size of 9 patients per intervention group was required to achieve at least a power of 80% to detect a mean difference of 5 mmHg in SBP measures, assuming a standard deviation (SD) of 3 mmHg (i.e., to detect an effect size of 1.67), at a significance level of .05.^39^ This number includes a potential dropout rate of about 10%.

#### Randomization

Randomization was unstratified, concealed and computer-generated by a senior statistician. This study was conducted as an open-label study since blinding was not possible for either healthcare professionals or participants.

#### Data Management

Given the involvement of multiple healthcare providers in lifestyle interventions, the REDCap (Research Electronic Data Capture)^40^ platform was used to streamline the management of data.

#### Statistical Analyses

Analyses were conducted using the statistical packages SAS (SAS University edition, version 2022). Participants characteristics and clinical measurements are expressed as Least-squared (LS) means ± SEM. The MIXED procedure was used to evaluate the time, group, and time × group interactions. Multiple regression analyses using the Generalized Linear Models (GLM) procedure were performed on the delta values of the interested variables to determine the potential influence of the changes in body composition and cardiometabolic markers on variations in SBP and DBP. The association between the medication status of participants and pre- and post-intervention variables of body composition and cardiometabolic health were tested using the general linear models implemented in the MIXED procedure and provided interactions of time, group, and time × group and age-adjusted LSmeans ± SEM data are presented. The results were considered statistically significant at *P* ≤ .05.

## Results

**Figure 2.**
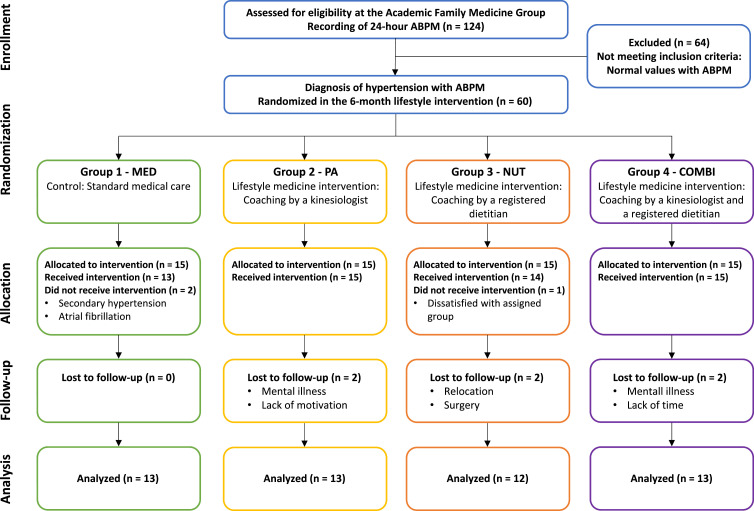
Flowchart of participants.

Figure 2 illustrates the flowchart of the study participants. As displayed in the flowchart, 3 participants did not start their allocated intervention due to other *de novo* diagnoses, that is, secondary HTN or atrial fibrillation, or due to dissatisfaction with group assignment. Therefore, 57 participants (MED: n = 13, PA: n = 15, NUT: n = 14; COMBI: n = 15) initiated the study. Fifty-one (51) participants (51/57, 89%) completed the 6-month follow-up. Six (6) patients did not complete the intervention due to *de novo* diagnosis such as secondary HTN or atrial fibrillation, surgery, or personal reasons and were therefore excluded from the analyses of the present study.

**Figure 3.**
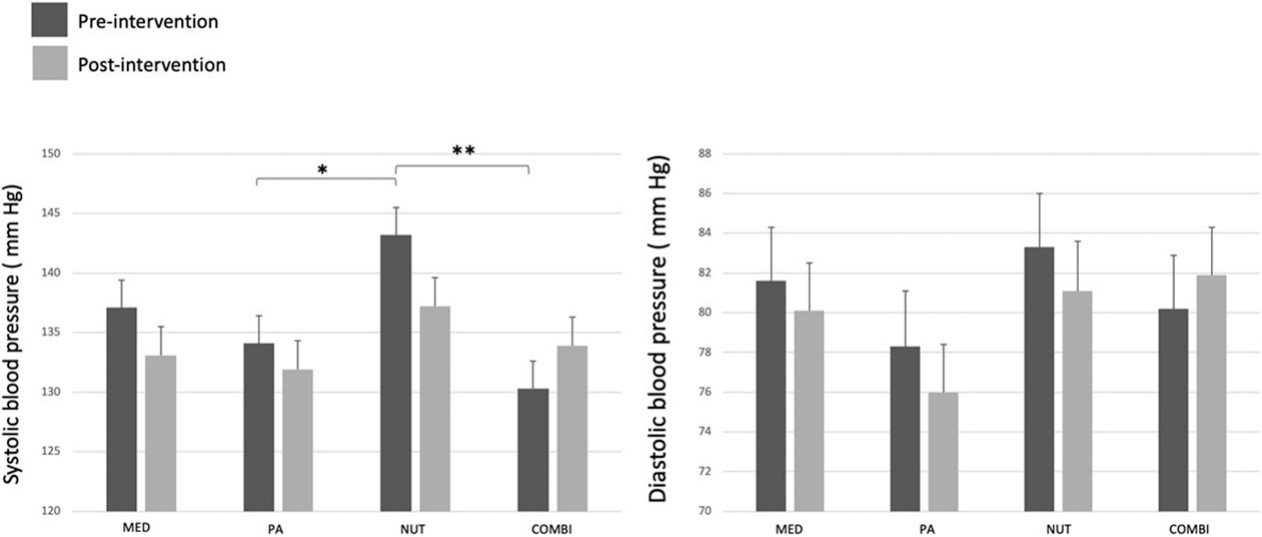
Changes in systolic and diastolic blood pressure pre- and post-intervention in each intervention group. Note: Values are presented as mean ± SD. SBP and DBP are overall 24-h ambulatory blood pressure monitoring mean. Statistical differences are presented as *when *P* ≤ .05 and as **when *P* < .01. Abbreviations: MED, standard medical care intervention group; PA, physical activity intervention group; NUT, nutrition intervention group (DASH diet); COMBI, combination of physical activity and nutrition interventions group.

Figure 3 illustrates the mean values of SBP and DBP measured with the 24-h ABPM before and after the 6-month intervention according to the intervention group. Results show that MED, PA, and NUT interventions induced a SBP decreased over time, while an increase was observed in the COMBI group where a significant group interaction (*P* = .04) was found in SBP, but no significant interactions for DBP (Table 1). It is noteworthy that there was a significant statistical difference in SBP between the NUT and the PA and COMBI groups at the beginning of the intervention (Figure 3).

**Table 1.** Descriptive Characteristics of Participants Pre- and Post-Intervention.

Variable	MED	PA	NUT	COMBI	Time Interaction	Group Interaction	Time × Group Interaction
	n = 13	n = 13	n = 12	n = 13	*P* value	*P* value	*P* value
Body weight (kg)							
Pre-intervention	83.7 ± 5.7	94.8 ± 5.7	84.0 ± 5.7	98.5 ± 5.7	.3	.2	.7
Post-intervention	84.2 ± 6.0	94.1 ± 6.0	82.5 ± 6.2	97.7 ± 6.0			
Body mass index (kg/m^2^)							
Pre-intervention	28.8 ± 62.0	32.7 ± 2.0	29.3 ± 2.0	35.0 ± 2.0	.3	.1	.7
Post-intervention	28.9 ± 2.0	32.5 ± 2.0	28.7 ± 2.1	34.8 ± 2.0			
Waist circumference (cm)							
Pre-intervention	98.7 ± 4.2	109.4 ± 4.2	103.4 ± 4.2	110.5 ± 4.2	.01*	.3	.01*
Post-intervention	100.8 ± 4.3	107.1 ± 4.3	98.1 ± 4.5	108.4 ± 4.3			
Visceral adipose tissue (cm^3^)							
Pre-intervention	1419 ± 184	1615 ± 184	1442 ± 191	1629 ± 184	.9	.8	.2
Post-intervention	1467 ± 205	1702 ± 205	1402 ± 214	16112 ± 214			
Systolic blood pressure (mm Hg)							
Pre-intervention	137.2 ± 2.3	134.1 ± 2.3	143.2 ± 2.4	130.3 ± 2.3	.07	.04*	.03*
Post-intervention	133.1 ± 2.4	131.9 ± 62.3	137.2 ± 2.4	133.9 ± 2.3			
Diastolic blood pressure (mm Hg)							
Pre-intervention	81.6 ± 2.7	78.3 ± 2.7	83.3 ± 2.8	80.2 ± 2.7	.2	.5	.3
Post-intervention	80.1 ± 2.4	76.0 ± 2.4	81.2 ± 2.5	81.9 ± 2.4			
HbA1c (%)							
Pre-intervention	5.2 ± .02	5.6 ± .02	5.9 ± .02	5.7 ± .02	.4	.5	.4
Post-intervention	5.6 ± .01	5.6 ± .01	5.7 ± .01	5.6 ± .01			
Total cholesterol (mmol/L)							
Pre-intervention	5.66 ± .31	4.83 ± .31	5.51 ± .32	5.26 ± .31	.7	.4	.4
Post-intervention	5.59 ± .27	5.07 ± .27	5.20 ± .28	5.25 ± .27			
Triglycerides (mmol/L)							
Pre-intervention	1.70 ± .10	1.27 ± .10	1.56 ± .10	1.73 ± .10	1.0	.7	.2
Post-intervention	1.44 ± .09	1.39 ± .09	1.82 ± .09	1.62 ± .09			
HDL-cholesterol (mmol/L)							
Pre-intervention	1.37 ± .20	1.49 ± .20	1.38 ± .21	1.30 ± .20	.05*	.5	.6
Post-intervention	1.38 ± .22	1.41 ± .22	1.33 ± .23	1.25 ± .22			
LDL-cholesterol (mmol/L)							
Pre-intervention	3.54 ± .26	3.01 ± .26	3.46 ± .27	3.17 ± .26	.4	.3	.1
Post-intervention	3.56 ± .26	3.01 ± .26	3.06 ± .27	3.27 ± .26			
Urinary sodium (mmol/L)							
Pre-intervention	93.4 ± 18.0	140.6 ± 15.2	100.8 ± 14.6	116.1 ± 15.2	.9	.1	.9
Post-intervention	102.1 ± 13.8	131.9 ± 13.7	101.1 ± 13.8	121.6 ± 13.8			
Estimated V̇O_2max_ (mLO_2_/kg/min)							
Pre-intervention	28.5 ± 2.2	27.8 ± 2.2	27.3 ± 2.3	30.1 ± 2.2	.2	.9	.08
Post-intervention	29.1 ± 2.2	28.3 ± 2.1	26.2 ± 2.2	26.6 ± 2.1			
Daily step count							
Pre-intervention	4447 ± 676	6206 ± 800	6691 ± 495	6339 ± 801	.3	.4	.3
Post-intervention	5971 ± 692	6573 ± 687	6537 ± 828	6884 ± 802			

Note: Values are presented as LS means ± SEM. SBP and DBP are overall 24-h ambulatory blood pressure monitoring mean. * when *P* ≤ .05.

Abbreviations: MED, standard medical care intervention group; PA, physical activity intervention group; NUT, nutrition intervention group (DASH diet); COMBI, combination of physical activity and nutrition interventions group; HbA1c, glycated hemoglobin; HDL, high-density lipoprotein; LDL, low-density lipoprotein.

Table 1 reports the pre- and post-intervention clinical characteristics of participants in each group. All interventions induced favorable changes in body composition, except in the MED group. Indeed, participants randomized in this latter group increased body weight, WC, and VAT, but not to a statistical extent. It is noteworthy that significant time and time × group interactions *(P* = .01) were found for WC between groups where WC increased in MED participants but reduced in PA, NUT, and COMBI participants. Analysis comparing the intervention groups vs MED group were further conducted by pooling PA, NUT, and COMBI groups together to compare changes over time (results not shown) and revealed a significant time × group interaction in WC (*P* = .003). Same favorable changes were found in cardiometabolic variables, that is, that the lifestyle interventions were leading to favorable changes or no change over time in PA, NUT, and COMBI groups, except for an increase in triglycerides in PA and NUT groups, an increase in total cholesterol in the PA group, and an increase in urinary sodium in the COMBI group. There were no significant interactions related to these changes, even when groups were combined in the additional analysis (results not shown). Interestingly, HDL-cholesterol decreased in all interventions (PA, NUT, COMBI) groups, with values above normal ranges.^41^ In the MED group, HbA1C, LDL-cholesterol, and urinary sodium values increased over time. Estimated V̇O_2max_ did not significantly differ over time, but participants in the COMBI group decreased their value by the end of the program. Daily step count increased in all groups except for the NUT group.

The multiple regression analyses considering changes in body composition and cardiometabolic variables with variations in SBP and DBP are presented in Table 2. Analyses were conducted on all groups combined, all interventions (PA, NUT and COMBI) pooled together, and on each intervention. Results show that lower values of total cholesterol are significantly associated with lower values of SBP in all interventions pooled (*P* = .04) and in the NUT group (*P* = .04) and a trend is observed when all groups are combined (*P* = .06). Lowered values of total cholesterol are also significantly associated with lowered values of DBP when all groups are combined (*P* = .01), in all interventions pooled (*P* = .01), in the PA group (*P* = .05) and in the NUT group (*P* = .03). Increased estimated V̇O_2max_ was significantly associated with lowered values of SBP in all groups combined (*P* = .05) and a trend was observed in all interventions pooled (*P* = .06). These changes in estimated V̇O_2max_ were also significantly associated with reduced DBP in groups combined (*P* = .01) and interventions pooled (*P* = .01), and a trend was observed in the PA group (*P* = .08). Lowered values of body weight (SBP: *P* = .009, DBP: *P* = .003) and BMI (SBP: *P* = .004, DBP: *P* = .003) were significantly associated with lower values of both SBP and DBP, and a trend in lower WC (*P* = .06) is observed with lower values of SBP in the NUT group. Increased daily step count was significantly associated (*P* = .05) and a trend in lowered LDL-cholesterol values (*P* = .08) was observed with reduced values of DBP in the NUT group. In the PA group, having lowered values of HbA1c was significantly associated with lowered values of DBP (*P* = .05).

**Table 2.** Multiple Regression Analyses of Changes in Cardiometabolic Markers and Changes in Systolic and Diastolic Blood Pressure.

Dependent Variables	Independent Variables	Total *R*^2^	*P* value
All 4 groups			
SBP	Total cholesterol	.07	.06
	Estimated V̇O_2max_	.08	.05
DBP	Total cholesterol	.13	.01
	LDL-cholesterol	.10	.02
	Estimated V̇O_2max_	.09	.03
All intervention groups			
SBP	Total cholesterol	.11	.04
	Estimated V̇O_2max_	.09	.06
DBP	Total cholesterol	.16	.01
	LDL-cholesterol	.13	.03
	Estimated V̇O_2max_	.10	.05
PA group			
DBP	HbA1c	.31	.05
	Total cholesterol	.32	.04
	Estimated V̇O_2max_	.25	.08
NUT group			
SBP	Body weight	.69	.0009
	Body mass index	.71	.0004
	Waist circumference	.31	.06
	Total cholesterol	.36	.04
DBP	Body weight	.60	.003
	Body mass index	.60	.003
	Total cholesterol	.38	.03
	LDL-cholesterol	.27	.08
	Daily step count	.41	.05

Note. All the interested cardiometabolic variables of the present study were included in the multiple regression analysis. The presented variables in Table 2 are the ones reaching statistical significance (*P* ≤ .05) in all 4 groups combined, in all intervention groups (PA, NUT, and COMBI) pooled together, and for each intervention.

Abbreviations: PA, physical activity intervention group; NUT, nutrition intervention group (DASH diet); SBP, systolic blood pressure; DBP, diastolic blood pressure; HbA1c, glycated hemoglobin; LDL, low-density lipoprotein.

**Figure 4.**
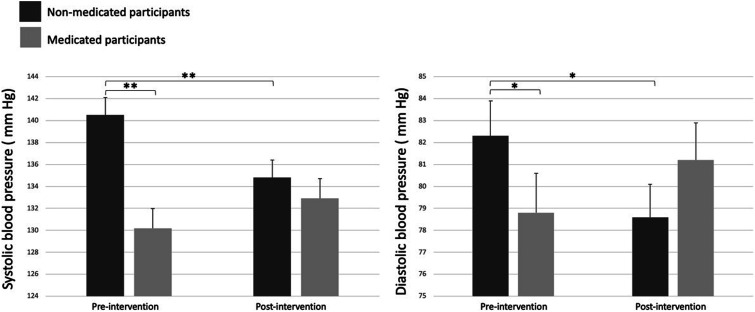
Changes in systolic and diastolic blood pressure pre- and post-intervention in non-medicated and medicated participants. Note: Statistical differences are presented as *when *P* ≤ .05 and as **when *P* < .01.

Sensitivity analyses were performed to determine the effect of a lifestyle intervention in NMP vs MP. Prior to the intervention, 28 participants were not taking antihypertensive medication and 23 were taking antihypertensive drugs (MED: n = 5, PA: n = 5, NUT: n = 7, COMBI: n = 6). Following the intervention, 2 participants were assigned antihypertensive drugs through the process (NUT: n = 2), 2 participants completely stopped their medication (PA: n = 1, NUT: n = 1), 5 participants decreased their antihypertensive drug dose (MED: n = 1, PA: n = 2, NUT: n = 1, COMBI: n = 1), and 13 had no change. Table 3 presents the characteristics and clinical measurements of NMP vs MP before and after the intervention and Figure 4 presents the changes in SBP and DBP in both groups over time. The results show a significant difference (*P* = .0009) in SBP and DBP between groups pre-intervention and significant time × group interaction in both BP indices (SBP: *P* = .009, DBP: *P* = .01). It is noteworthy that pre-intervention SBP values were significantly (*P* = .001) higher in NMP and that DBP values were also higher in this groups compared to their MP counterparts. A significant difference in HbA1c between groups was also observed (*P* = .04). The time × group interaction was statistically significant for urinary sodium with MP showing greater values at both times but increasing over time and NMP decreasing by the end of the intervention program.

**Table 3.** Descriptive Characteristics of Non-Medicated Participants (NMP) vs Medicated Participants (MP) Pre- and Post-intervention.

Variable	Pre-Intervention		Post-Intervention		Time Interaction	Group Interaction	Time × Group Interaction
	NMP	MP	NMP	MP			
	(n = 28)	(n = 23)	(n = 29)	(n = 22)	*P* value	*P* value	*P* value
Age (years)	50.3 ± 2.3	58.8 ± 2.6					
Body weight (kg)	90.6 ± 3.8	90.0 ± 4.4	88.2 ± 3.9	91.7 ± 4.5	.3	.6	.4
Body mass index (kg/m^2^)	30.9 ± 1.4	32.2 ± 1.6	30.4 ± 1.4	32.4 ± 1.6	.2	.8	.4
Waist circumference (cm)	105.1 ± 3.0	106.2 ± 3.4	102.2 ± 2.9	105.7 ± 3.4	.02*	.9	.5
Visceral adipose tissue volume (cm^3^)	1468 ± 125	1606 ± 145	1497 ± 141	1612 ± 159	.9	.6	.2
SBP (mmHg)	140.5 ± 1.6	130.2 ± 1.8	134.8 ± 1.6	132.9 ± 1.8	.2	.0009**	.009**
DBP (mmHg)	82.3 ± 1.6	78.8 ± 1.8	78.6 ± 1.5	81.2 ± 1.7	.3	.0009**	.01*
HbA1c (%)	5.5 ± 0.1	5.9 ± 0.1	5.5 ± .07	5.7 ± .08	.4	.04*	.2
Total cholesterol (mmol/L)	5.32 ± .22	5.30 ± .25	5.37 ± .18	5.16 ± .21	.8	.8	.9
HDL-cholesterol (mmol/L)	1.45 ± .06	1.31 ± .07	1.37 ± .05	1.30 ± .06	.06	.8	.6
LDL-cholesterol (mmol/L)	3.29 ± .15	3.30 ± .17	3.33 ± 016	3.10 ± .18	.4	.5	.5
Triglycerides (mmol/L)	1.28 ± .17	1.95 ± .19	1.47 ± .18	1.60 ± .20	.8	.2	.2
Urinary sodium (mmol/L)	111.9 ± 10.1	117.9 ± 15.9	100.6 ± 9.04	130.5 ± 9.7	.6	.3	.05*
Estimated VO_2max_ (mL/kg/min)	28.6 ± 1.2	28.3 ± 1.4	28.0 ± 1.2	27.0 ± 1.4	.2	.6	.7
Daily step count	6140 ± 2622	5606 ± 2528	6889 ± 2118	5883 ± 2821	.2	.5	.6

Note. Values are presented as LS means ± SEM. SBP and DBP are overall 24-h ambulatory blood pressure monitoring mean. *When *P* ≤ .05, ** when *P* < .01.

Abbreviations: NMP, non-medicated participants; MP, medicated participants; SBP, systolic blood pressure; DBP, diastolic blood pressure; HbA1c, glycated hemoglobin; HDL, high-density lipoprotein; LDL, low-density lipoprotein.

Use of over-the-counter drugs was documented due to its well-known association with increased BP values.^42^ In the MED group, 2 participants used over-the-counter drugs (acetaminophen and antihistamine), 3 participants in the PA group used this type of drugs (muscle relaxant, chloroquine, and ibuprofen), 3 participants in the NUT group used acetaminophen, and 1 participant in the COMBI group used antihistamine.

## Discussion

The main goals of this randomized controlled pilot study were to evaluate the feasibility and effect of a 6-month interdisciplinary lifestyle program among stage 1 hypertension patients, focusing on changes in SBP and DBP in primary care settings. Additionally, it aimed to investigate the relationship between changes in SBP and DBP and variations in VAT, CRF, and cardiometabolic markers. Furthermore, the study aimed to document changes in SBP, DBP and cardiometabolic parameters in hypertensive patients receiving antihypertensive medication compared to those who were not. The results of this study suggest that adopting a healthy lifestyle seems feasible in the context of HTN management in primary care settings since 89% (51/57) of patients who received their allocated intervention completed the whole program. Patients with HTN adopting the DASH diet recommendations seem to benefit from significant SBP reduction and, not to a statistical extent, DBP reduction. It is noteworthy that BP indices in the NUT group were initially higher and this elevation remained at the end of the intervention compared to the 3 other intervention groups, which provided favorable opportunity for the NUT group to significantly improve BP values by the end of the intervention. Reductions in SBP and DBP were also observed in the PA and MED groups. Unexpectedly, individuals randomized in the COMBI group depicted both SBP and DBP slight increase over time. Despite this small rise, the COMBI group still exhibited lower SBP values compared to the NUT groups at the end of the intervention. The additional analysis pooling all the intervention groups together to compare to the control group did not reveal further significant statistical differences in SBP and DBP variations over time. Therefore, in the context of the present study, one cannot conclude that one approach is better than another. However, the multivariable analysis revealed interesting associations between variations in SBP and changes in total cholesterol and estimated V̇O_2max_ as well as variations in DBP and total cholesterol, LDL-cholesterol, and estimated V̇O_2max_, suggesting that improving cardiometabolic profile by adopting a healthy lifestyle may induce favorable changes on BP indices. This aligns with the current literature, which demonstrates a relationship between a favorable lipid profile^43^ and high fitness^44^ as well as BP indices. It is important to highlight that interdisciplinary lifestyle interventions appear to enhance BP indices in most patients diagnosed with stage 1 HTN. This investigation not only demonstrates the feasibility of implementing lifestyle interventions in real-life, interdisciplinary primary care settings and their beneficial impact on cardiometabolic status, but also that patients exhibit compliance with these types of interventions.

### Effect of Lifestyle Interventions on Body Composition and Cardiometabolic Parameters

#### NUT Group

Adopting a healthy lifestyle is well-recognized for its beneficial effect on HTN prevention and management, including adopting a healthy diet, reducing sodium intake and alcohol consumption, and practicing regular physical activity.^2,45,46^ The DASH diet, designed for prevention and treatment of HTN, has shown efficacy,^30,31^ with some studies indicating that individuals with initially high BP values experience the most significant reductions in both SBP and DBP upon adopting this dietary approach.^47^ This aligns with our results where participants in the NUT group, initially displaying the highest BP values, decreased them post-intervention. Multivariable regression analyses showed that SBP and DBP variations were associated with changes in body weight, BMI, and total cholesterol in the NUT group. The current literature demonstrates the beneficial relationship between weight loss and BP lowering,^48,49^ indicating that a weight loss of ∼8 kg is associated with a clinically significant reduction in both SBP and DBP of 7-10 mm Hg and of 6-7 mm Hg, respectively.^50^ The impact of the DASH diet on weight loss^51,52^ may also have contributed to the improvement of SBP and DBP in the NUT group. Furthermore, it well-documented that diet intervention induces favorable changes in body composition.^53^ Additionally, the positive effect of the DASH diet on total cholesterol and LDL-cholesterol^30^ suggests that targeting interventions for both HTN and hypercholesterolemia could favorably improve overall cardiovascular health.^54,55^

#### PA Group

A body of evidence suggests a positive relationship between BP lowering and exercice,^56,57^ aligning with our findings. The American Heart Association experts’ systematic review and a meta-analysis indicated that endurance aerobic training, dynamic resistance training and isometric resistance training all lower SBP and DBP, while combined exercise only affects DBP.^58^ In the present study, the promoted PA guidelines were to perform at least 4 sessions of aerobic activities for a duration between 30 and 60 min and at least 2 strength training sessions weekly, as well as reaching a 10,000 daily steps count. This combined exercise recommendation potentially influenced the relationship between PA and BP variations. The focus on a specific type of exercise appears to be more effective in influencing both SBP and DBP variations in patients with HTN, emphasizing the importance of promoting this approach for achieving anticipated BP reduction with exercise. Furthermore, participants in the PA and COMBI groups were meeting once a month with the kinesiologist. Even if education on intensity of physical activity practice was given to patients, the perception of intensity is known to differ from one individual to another. Therefore, the stimulus of an increased intensity might not have been sufficient in participants to which physical activity was prescribed to induce marked improvements in estimated V̇O_2max_. Multiple regression analyses revealed significant associations between variations in DBP and changes HbA1c and total cholesterol and a trend with changes in estimated V̇O_2max_ in the PA group. This is in accordance with other studies that demonstrated the favorable association of HbA1c,^59,60^ total cholesterol,^61^ and V̇O_2max_^62,63^ with increased regular physical activity practice.

#### COMBI Group

In the COMBI group, both SBP and DBP increased from pre- to post-intervention, contrary to expectations given the well-known positive impact of a healthy diet and exercise on BP reduction. While some studies reported inconclusive effects of combined interventions on BP,^64^ a similar randomized controlled trial conducted among Korean adults (n = 85) demonstrated significant 24-h mean reduction in both SBP (−5.4 ± 8.8 mm Hg) and DBP (−4.1 ± 6.8 mm Hg) with a combination of DASH diet and exercise.^65^ Possible factors contributing to the unexpected results in the COMBI group include the presence of adverse responders. Adverse responders, defined as individuals showing no change or unfavorable variation to an intervention, have been reported in previous research.^66,67^ The medication status of COMBI participants, with more than half (6/13, 54%) taking antihypertensive medication initially and the higher body weight might also have influenced the response to the intervention. Medication compliance could also have affected the elevated BP measured in the COMBI group. The complex nature of individual responses to lifestyle interventions involves genetic, behavioral, metabolic, physiological, and environmental factors. Obesity is known to be related to high BP values.^8^ Participants assigned to the COMBI group, who were initially obese individuals (BMI ≥35 kg/m^2^), maintained this BMI at the end of the intervention. They also demonstrated the smallest increase in their daily step count, suggesting a likely smallest engagement in daily physical activity. Taken together, this may have contributed to the unfavorable BP variations. The present study highlights the importance of personalized healthcare approaches to address individual needs and optimize intervention effectiveness, emphasizing a step toward precision medicine through the identification and understanding of adverse responders.

#### MED Group

Participants in the MED group displayed favorable variations in both SBP and DBP. A registered nurse provided advice on healthy diet and exercise to reduce BP indices at the beginning of the intervention and was meeting with them monthly for BP monitoring. The consistent follow-up with a health professional may have positively influenced the participants’ behavior.^68^ It is noteworthy that they slightly increased their estimated V̇O_2max_ and, although not to a statistically significant extent, their daily step count even though not reaching the 10,000 steps per day recommended. This beneficial health behavior change may be attributed to the Hawthorne effect, described as intentional behavioral modification resulting from study participation and monitoring,^69^ similarly to the placebo effect in pharmacological studies.

##### Impact of Lifestyle Medicine on Medication Management

In a secondary analysis comparing the impact of lifestyle interventions in NMP vs MP, noteworthy results emerged. Lifestyle interventions significantly induced SBP and DBP reductions in the NMP group, while the MP group experienced an increase in both BP indices. Furthermore, some participants completely stopped taking their antihypertensive medication and others decreased their medication dose by the end of the intervention, indicating that lifestyle interventions favorably influence BP leading to a free-antihypertensive-medication way of living. Various health organizations, including the International Society of Hypertension,^13^ the American Medical Association,^14^ and Hypertension Canada,^15^ emphasize the substantial impact of lifestyle interventions in the prevention and management of HTN. The ESC/ESH recommends prioritizing lifestyle interventions for 3 to 6 months before prescribing antihypertensive medication in patients with mild HTN.^13^ This secondary analysis demonstrated that participants initially not taking medication, despite exhibiting higher BP values initially, achieved the lowest BP values by the end of the intervention, supporting the effectiveness of lifestyle interventions in HTN management. Notably, 5 participants reduced their medication dose and two no longer required medication by the end of the 6-month intervention, highlighting the potential of deprescription. These findings hold public health significance, reinforcing that adopting a healthy lifestyle not only reduces BP but also enhances cardiometabolic health contributing to overall cardiovascular benefits.

##### Over-the-Counter Drugs and Blood Pressure

Participants reported using over-the-counter drugs to alleviate muscle soreness after exercise sessions. The collaboration with a pharmacologist revealed that some over-the-counter drugs, that is, ibuprofen and muscle relaxants can contribute to BP elevation. Therefore, higher BP values by the end of intervention in participants randomized in both PA and COMBI groups might have been influenced by using over-the-counter drugs, specifically if participants were not used to exercise prior to being involved in the program and experiencing muscle soreness. This aligns with findings of other investigations indicating an increase in BP with over-the-counter drug and natural products use, particularly in individuals with HTN.^42^ Monitoring of over-the-counter drugs by healthcare professionals in the context of HTN management and patient education on the detrimental effect of these medications on BP reduction are crucial for a comprehensive assessment of the patient profile.

##### Strengths and Limitations

The present study has many strengths. This structured interdisciplinary randomized study conducted in a real-life context, demonstrated the beneficial effects of lifestyle interventions on HTN management and cardiometabolic health markers. It also highlighted the feasibility of implementing these interventions in primary care settings. The randomization of participants ensured equal opportunities for assignment to one of the four intervention groups, reducing sampling biases. Employing various gold-standard tools such as the ABPM, computed tomography scans and exercise testing, allowed a more comprehensive characterization of the cardiometabolic profile of the participants. For instance, prior to the intervention, 124 patients were diagnosed with HTN and referred based on office BP measurement. After undergoing a 24-h ABPM, 64 of them were excluded from the research project as they did not display HTN. This justifies the use of gold-standard tools, as relying solely on physician’s office BP measurements could lead to overdiagnosis and over-prescription. Clinical adjustments, such as monitoring over-the-counter medication use with a tool developed by the primary care pharmacist, and modifying the positioning during BP measurement (e.g., seated position with feet on the ground and arm resting to heart level)^24^ were emphasized in the context of the study. Furthermore, the participation of a patient partner facilitated the exploration of novel approaches to integrate and facilitate lifestyle interventions into clinical settings and to initiate new research projects. Examples include the creation of a “magnet 101 sodium” to improve understanding of reading food labels and assessing salt quantities in food, as well as the incorporation of connected devices to optimized PA motivation.

This study also has some limitations. Our findings are based on data from a small sample size with limited cultural diversity, which limits the generalization of conclusions. Highlighting the importance of evaluating adherence to interventions via questionnaires and monitoring medication compliance is crucial to ensure that the prescribed intervention intensity is achieved and leads to the expected changes. For instance, measuring physical activity intensity may likely result in greater improvements in V̇O_2max_. Furthermore, these measures are essential for gaining a deeper understanding of patient profiles, facilitating the implementation of precision medicine tailored to their needs. Larger, longitudinal, and culturally diverse, geographically and gender-diverse studies are needed to better understand the impact of lifestyle medicine interventions on the management of HTN in primary care settings.

## Conclusion

The findings of this 6-month randomized study suggest that structured interdisciplinary lifestyle medicine interventions are feasible in a real-life primary care setting context in stage 1 HTN patients. Favorable changes in body composition and cardiometabolic profile induced by the adoption of a healthy lifestyle seem to influence variations in both SBP and DBP over time. Lifestyle interventions seem to allow the avoidance of antihypertensive medication prescription as preventive care in some patients with HTN.

Considering the escalating global prevalence of HTN and the associated increase in concordant healthcare costs, there is an imperative need to prioritize prevention and management of HTN. Despite compelling evidence supporting favorable effects of lifestyle interventions in both primary prevention and treatment of HTN, challenges remain in integrating this knowledge into regular medical practice. Efforts to enhance non-pharmacological approaches are needed to improve HTN preventive care and management guidelines. We also believe that long-term randomized controlled trials examining plant-based diets, the DASH diet, and other diets on HTN will help refine our understanding of the relationship between diet and cardiovascular disease. Additional research, particularly in larger and more diverse cohorts, is essential to better understand the impact of lifestyle interventions in primary care settings. Conducting research on adverse responders and implementing precision medicine are essential to ensure that lifestyle interventions are centered around the individual.

**CME/CE Article Quiz**

American College of Lifestyle Medicine (ACLM) members can earn FREE CME/CE credit by reading this approved CME/CE article and successfully completing the online CME/CE activity. Non-members can earn CME/CE for $40 per article. Visit lifestylemedicine.org to join the ACLM.

**Instructions.**

1. AJLM CME/CE Articles and Quizzes are offered online only through the American College of Lifestyle Medicine and are accessible at lifestylemedicine.org/store. ACLM Members can enroll in the activity, complete the quiz, and earn this CME/CE for free. Non-members will be charged $40 per article.
2. A Passing score of 80% or higher is required in order to be awarded the CME/CE credit.

## Supplemental Material

Supplemental Material - Impact of Lifestyle Medicine Interventions on the Management of Systemic Hypertension in Primary Care: A Canadian Randomized Controlled Trial Supplemental Material for Impact of Lifestyle Medicine Interventions on the Management of Systemic Hypertension in Primary Care: A Canadian Randomized Controlled Trial by Elisa Marin-Couture, Julie-Alexandra Moulin, Anne-Sophie Thibault, Paul Poirier, Jean-Pierre Després, Anette Gallant, Vincent Lamarre, Natalie Alméras, Isabelle Lemieux, Christian Chabot, Maria-Cecilia Gallani, Marie-Eve Piché, Benoit J. Arsenault, Angelo Tremblay, Jean-Sébastien Paquette and Caroline Rhéaume in American Journal of Lifestyle Medicine.
