# Supplementary material for: Impact of Lifestyle Medicine Interventions on the Management of Systemic Hypertension in Primary Care: A Canadian Randomized Controlled Trial
Source: Am J Lifestyle Med. 2024 Apr 4;18(5):703–20. doi: 10.1177/15598276241242013 (PMC12032512; doi:10.1177/15598276241242013)
Supplement: Supplemental Material - Impact of Lifestyle Medicine Interventions on the Management of Systemic Hypertension in Primary Care: A Canadian Randomized Controlled Trial [file sj-pdf-1-ajl-10.1177_15598276241242013.pdf]

American Journal of Lifestyle Medicine (AJLM) CME/CE Article Quiz Volume 18, Issue 5

Impact of Lifestyle Medicine Interventions on the Management of Systemic Hypertension in Primary Care: A Canadian Randomized Controlled Trial

American College of Lifestyle Medicine (ACLM) members can earn FREE CME/CE credit by reading this approved CME/CE article and successfully completing the online CME/CE activity. Non-members can earn CME/CE for $40 per article. Visit lifestylemedicine.org to join the ACLM.

Instructions

1. AJLM CME/CE Articles and Quizzes are offered online only through the American College of Lifestyle Medicine and are accessible at lifestylemedicine.org/store. ACLM Members can enroll in the activity, complete the quiz, and earn this CME/CE for free. Non-members will be charged $40 per article. This CME/CE opportunity is available and expires September 2, 2027.
2. A Passing score of 80% or higher is required in order to be awarded the CME/CE credit.

Article and Authors: Impact of Lifestyle Medicine Interventions on the Management of Systemic Hypertension in Primary Care: A Canadian Randomized Controlled Trial - Elisa Marin-Couture, Julie-Alexandra Moulin, Anne-Sophie Thibault, Paul Poirier, Jean-Pierre Després, Anette Gallant, Vincent Lamarre, Natalie Alméras, Isabelle Lemieux, Christian Chabot, Maria-Cecilia Gallani, Marie-Eve Piché, Benoit J. Arsenault, Angelo Tremblay, Jean-Sébastien Paquette, Caroline Rhéaume, 2024 (sagepub.com)

Learning Objectives: After studying the article, you will be able to:

1. Describe the dietary and physical activity interventions used to reduce blood pressure
2. State the minimum difference in systolic blood pressure (SBP) this study was designed to detect
3. Determine which interventions reduced SBP and diastolic blood pressure (DBP)
4. Compare the effect of lifestyle interventions on blood pressure in participants who were taking anti-hypertensive medications and those who were not on these medications

Accreditation and Joint Providership Statement:

In support of improving patient care, this activity has been planned and implemented by RUSH University Medical Center and American College of Lifestyle Medicine. RUSH University Medical Center is jointly accredited by the Accreditation Council for Continuing Medical Education (ACCME), Accreditation Council for Pharmacy Education (ACPE), and the American Nurses Credentialing Center (ANCC), to provide continuing education for the healthcare team.

Designation Statement:

For Medicine: Rush University Medical Center designates this journal-based CME activity for a maximum of 1.00 *AMA PRA Category 1 Credit(s)™*. Physicians should claim only credit commensurate with the extent of their participation in the activity.

Successful completion of this CME activity, which includes participation in the evaluation component, enables the learner to earn credit toward the CME of the American Board of Surgery's Continuous Certification program. It is the CME activity provider's responsibility to submit learner completion information to ACCME for the purpose of granting ABS credit.

For Nursing: Rush University Medical Center designates this journal-based CME activity for a maximum of 1.00 nursing contact hour(s).

For Pharmacy: Rush University Medical Center designates this knowledge-based journal-based CME activity for a maximum of 1.00 contact hour(s) for pharmacists.

For Psychologists: Rush University Medical Center designates this journal-based CME activity for 1.00 CE credits in psychology.

For Dieticians: This journal-based CME activity has been approved by the Commission on Dietetic Registration for 1.00 CPEUs.

For Social Work: As a Jointly Accredited Organization, Rush University Medical Center is approved to offer social work continuing education by the Association of Social Work Boards (ASWB) Approved continuing education (ACE) program. Organizations, not individual courses, are approved under this program. Regulatory boards are the final authority on courses accepted for continuing education credit. Social workers completing this course receive 1.00 general continuing education credits.

For Physical Therapy or Occupational Therapy: Rush University Medical Center is an approved provider for physical therapy (216.000378) and occupational therapy (224.000220) by the Illinois Department of Professional Regulation.

Rush University Medical Center designates this journal-based CME activity for 1.00 continuing education credits.

By participating in this activity, you allow your learner data to be shared with the ACCME and the appropriate certifying board.

ABLM MOC: The American Board of Lifestyle Medicine has approved 1.0 maintenance of certification credits for this learning activity.

American Journal of Lifestyle Medicine (AJLM) CME/CE Article Quiz Volume 18, Issue 5

Question 1: Which of the following was part of the physical activity goals for the physical activity and combination groups?

a. Achieving at least 7,500 daily steps
b. Engaging in aerobic activities at least 5 days per week
c. Engaging in aerobic activity sessions of 30-60 minutes duration
d. Engaging in 3 strength training sessions per week

Question 2: Which of the following groups experienced an increase in systolic blood pressure (SBP) and diastolic blood pressure (DBP)?

a. Combination of physical activity and nutrition interventions
b. Nutrition intervention
c. Physical activity intervention
d. Standard medical care intervention

Question 3: This study aimed to have a minimum sample size of 9 patients per intervention group. This would yield a power of 80% to detect a mean difference of ______ mm Hg in SBP measures.

a. 1
b. 3
c. 5
d. 7

Question 4: Which of the following dietary approaches was used in the nutrition intervention?

a. Dietary Approach to Stop Hypertension (DASH) Diet
b. Mediterranean Diet
c. Portfolio Diet
d. Vegan Diet

Question 5: Which of the following statements best represents the impact that lifestyle interventions had in participants who were medicated with anti-hypertensive medications (MEDS) versus participants who were not on anti-hypertensive medications (NOMEDS)?

a. Lifestyle interventions failed to reduce blood pressure in any participants (MEDS and NO MEDS)
b. Lifestyle interventions induced reductions in blood pressure in participants who were on MEDS but *not* in patients who were on NOMEDS
c. Lifestyle interventions induced reductions in blood pressure in participants who were on NO MEDS but *not* in patients were on MEDS
d. Lifestyle interventions induced similar changes in blood pressure in all participants (MEDS and NO MEDS)
